# Biostimulants from *Rosa alba* L. optimize growth and physiological processes of *Zea mays* L

**DOI:** 10.3389/fpls.2026.1731231

**Published:** 2026-04-01

**Authors:** Fayaz Asad, Abida Zeb, Sabrina Shahid, Sarir Ahmad, Michael Wittenberg, Mihail Angelov, Tabassum Yaseen, Akhtar Ali, Tsanko Gechev, Sajid Ali

**Affiliations:** 1 Department of Botany, Bacha Khan University, Charsadda, Pakistan; 2 Department of Botany, Women Campus Charbagh, University of Swat, Dera Ismail Khan, Pakistan; 3 Department of Forestry, University of Agriculture, Dera Ismail Khan, Pakistan; 4 Department Molecular Stress Physiology, Center of Plant Systems Biology and Biotechnology, Plovdiv, Bulgaria; 5 Global Plant Stress Research Center, Konkuk University, Seoul, Republic of Korea; 6 Department of Molecular Biology, Plovdiv University, Plovdiv, Bulgaria; 7 Department of Horticulture and Life Science, Yeungnam University, Gyeongsan, Republic of Korea

**Keywords:** biostimulants, maize, photosynthetic pigments, *Rosa alba*, *Zea mays*

## Abstract

**Introduction:**

Contemporary agriculture encounters significant challenges due to the excessive application of synthetic pesticides and fertilizers, leading to increased environmental and health issues. Plant-based biostimulants represent a viable solution for improving crop productivity with reduced ecological impacts.

**Methods:**

In this work, we examined how the growth and metabolic responses of *Zea mays* L. seedlings were affected by dose-dependent powdered leaf and stem biomass of *Rosa alba* L. (5, 10, and 15 g.kg^−1^ of soil).

**Results:**

The findings indicated that increased powdered stem biomass doses, specifically 10 g and 15 g, markedly improved growth factors including root length, leaf quantity, and shoot length, whereas a little influence was observed in root quantity. The 10 g treatment was identified as the most balanced dosage, facilitating significant root and shoot development, whereas the 15 g treatment predominantly enhanced root elongation. Enhancements in photosynthetic pigments, such as chlorophyll a, b, total chlorophyll, and carotenoids, observed under the 10 g and 15 g treatments, suggest increased photosynthetic efficiency and improved photoprotection mechanisms. Biochemical analysis indicated increase in protein and sugar levels with elevated treatment doses, demonstrating improved metabolic activity and nutrients assimilation. In contrast, proline content, recognized as a stress marker, exhibited a progressive decrease with higher powdered biomass concentrations, indicating diminished stress levels and enhanced physiological stability. Principal Component Analysis (PCA) identified photosynthetic pigments and sugar content as the primary factors contributing to treatment-induced variability, with proline serving as a specific indicator of stress response.

**Discussion:**

This study presents strong evidence that *R. alba* powdered biomass, especially at optimized doses, function as new effective natural growth enhancers. These *R. alba* biostimulants enhance plant growth, photosynthetic capacity, and metabolic stability, while also mitigating stress, thereby providing a sustainable and environmentally friendly alternative to synthetic agrochemicals.

## 1 Introduction

Allelopathy is a biological phenomenon in which plants release secondary metabolites, known as allelochemicals, that affect the growth, germination, or development of neighboring plants Bachheti et al., 2020; Chen et al., 2017). Interactions may be classified as either inhibitory or stimulatory, depending on the chemicals’ composition and concentration. Recent studies indicate a shift from traditional usage of allelopathy for weed control (Wang et al., 2008), to its potential role as a naturally occurring biostimulant in sustainable agricultural practices. Plant-based biostimulants are increasingly recognized for their environmentally friendly characteristics and their capacity to enhance crop growth, resilience, and productivity, while avoiding the adverse effects linked to synthetic agrochemicals. Organic goods and ecological farming have emerged as a result of growing awareness, underscoring the need of safer and more ecologically friendly methods (Kong et al., 2024). Plant-based allelochemicals, in particular plant biostimulants, show great promise since they are nontoxic, biodegradable, enhance plant growth, stimulate plant development, and can increase crop resistance to a variety of challenges (Di Sario et al., 2025; Kostina-Bednarz et al., 2023; Tlak Gajger and Dar, 2021). In contrast with fertilizers, which supplement missing nutrients, the biostimulants activate particular processes related to plant growth or/and stress responses. According to the EU definition, the biostimulants are “stimulating plant nutrition processes independently of the product’s nutrient content with the sole aim of improving one or more of the following characteristics of the plant or the plant rhizosphere: (a) nutrient use efficiency; (b) tolerance to abiotic stress; (c) quality traits; (d) availability of confined nutrients in soil or rhizosphere” (Gonçalves et al., 2025). The majority of the plant biostimulants are based on seaweed extracts, but there are also biostimulants from other plants, microbial biostimulants, protein hydrolysates, and other small organic molecules (Sujeeth et al., 2022).

*Rosa alba* L., a member of the Rosaceae family, is a medicinal and ornamental plant known for its diverse biological properties. Traditionally, it has been utilized as a laxative, for treating eye infections, and as a significant component in fragrant oils and skincare products (Basim and Basim, 2003; Labban and Thallaj, 2020; Verma et al., 2020). Its phytochemical richness contributes to notable antibacterial, antifungal, antioxidant, anti-viral, and anti-genotoxic properties, providing it a subject of increasing scientific interest (Alom et al., 2021; Gateva et al., 2022, 2020; Valcheva et al., 2024; Vilhelmova-Ilieva et al., 2021). *R. alba* is widely cultivated in the agricultural areas of Asian countries such as Pakistan, Europe (Bulgaria, Scandinavia), and North America (Canada) (Dobreva et al., 2021; Georgieva et al., 2019). In the study area, *R. alba is* commonly cultivated along agricultural field boundaries for aesthetic purposes and practical functions, notably serving as a natural defense against grazing animals. The close distance to cereal crops such as *Zea mays* (maize) suggests potential biochemical interactions through exudates from the roots, leachates, or decomposing plant material (Zeev et al., 2025). Although *R. alba* is widely utilized and exhibits promising bioactivity, its potential of organic soil amendment effects on staple food crops have not been thoroughly investigated. To fill this gap, we conducted both laboratory and field tests to examine the growth-promoting effects *R. alba* leaf and stem powder biomass on *Z. mays* through key morphological and biochemical characteristics. Our work hypothesis was that the *R. alba* leaf and stem powder biomass could promote growth of maize and positively influence biochemical parameters and stress markers, which in turn could indicate future use of *R. alba* as growth promoter in cereals. The use of small quantities of *R. alba* to improve plant growth is fully aligned with the EU definition of biostimulants.

## 2 Materials and methods

### 2.1 Preparation of *R. alba* leaf and stem powder

*R. alba* L. plants were gathered with local permission from Tehsil Dargai, District Malakand, Akbar Abad Kopar. After collection the plants were air dried in room temperature (22–28 °C) for 15 days. Using an electric grinder (RAF Electric Grinder, Model R−7132, Pakistan), dry materials (leaves and stems) were crushed into a fine powder. The estimated particle size was ~0.4–1.0 mm, although no sieve analysis was conducted. The stem and leaf powdered biomass was stored in clean, labeled plastic bags at room temperature for further use. The powdered material was applied to soil in pots at three treatment levels: 5, 10, and 15 g.kg^−1^ of soil.

### 2.2 GC-MS and UHPLC-MS analysis to identify metabolites present in the *R. alba* material used for the biostimulant treatment

As only partial phytochemical profile of *Rosa alba* biomass was characterized in previous research (Georgieva et al., 2019; Verma et al., 2020), here we have performed a detailed GC-MS and UHPLC-MS analysis to identify the primary and secondary metabolites present in the *R. alba* material used for the biostimulant treatment. Metabolites were extracted from 20 mg of air-dried *Rose alba* tissue from either stems or leaves. The tissue was ground to a fine powder and extracted with 1 ml of LCMS grade water spiked with 4 µg.ml^−1^ ribitol and 1.25 µg/ml isovitexin. After incubation on an orbital shaker at 30°C for 30 min. at 1200 rpm, the samples were sonicated in a sonication water bath for 15 min. Finally, they were centrifuged at 10,000 g for 10 minutes after which the supernatant was removed and filtered through a 0.2 µm filter.

Derivatization for the GC-MS was carried out as in Lisec et al. (2006) on 100 µl of dried metabolite extract using 20 mg/ml methoxyamine hydrochloride in pyridine and trimethylsilyl-*N*-methyl trifluoroacetamide. Derivatized extracts were analyzed on a TSQ9000 GCMS (Thermo Fisher Scientific) following a one microliter injection. Helium was used as carrier gas at a constant flow rate of 2 ml.sec^−1^ and gas chromatography was performed on a 30-m DB-35 column with, 0.32 mm inner diameter and 0.25 μm film thickness (Agilent Technologies). The injection temperature was 230 °C and the transfer line and ion source temperatures were set to 250 °C. The initial temperature of the oven (85 °C) increased at a rate of 15 °C min^−1^ up to a final temperature of 360 °C. After a solvent delay of 180 sec, mass spectra were recorded at 20 scans.sec^−1^ with 70–600 *m*/*z* scanning range. The chromatograms were processed in Xcalibur v.4.1.31 and peaks were annotated to an internal library of over 100 standards recently analyzed on the same instrument. In addition, prominent peaks not present in the internal library were manually annotated by matching spectra to the NIST 14 spectral library. Metabolite annotations were ranked by confidence as follows: annotations confirmed by authentic standards were assigned a value of 1; annotations supported by matching spectra and consistent retention times but lacking standards were assigned a value of 2; and putative annotations were assigned a value of 3.

In addition to the GC-MS analysis, samples were also analyzed on a UHPLC-MS system (ACQUITY UPLC I-Class PLUS, SYNAPT XS; Waters, USA). Chromatographic conditions were as follows: BEH C18 column (1.7 µm, 2.1 mm x 100 mm; Waters, USA), kept at 40 °C. Sampler temperature set at 15 °C. Mobile phase A 0.1% formic acid (LiChropur, Merck)-water (LiChrosolv, Merck), and phase B 0.1% formic acid-acetonitrile (Chromasolv, Honeywell). Flow rate at 0.4 mL.min^−1^. Gradient: 0–1 min, 1% B; 1–11 min, 1-40% B; 11–13 min, 40-70% B; 13–15 min, 70-99% B; 15–16 min, 99% B; 16–17 min, 99-1% B; 17–20 min, 1% B. Injection volume 1 µL. Q-TOF mass spectrum conditions: an electrospray ion source (ESI) was used. Capillary voltage 2.5 kV, sampling cone voltage 40 V, source temperature 120 °C, desolvation temperature 250 °C. Cone gas flow 50 L.h^−1^, desolvation gas flow 600 L.h^−1^ and nebulizer gas flow 6.5 bar. The detector was set in negative mode, resolution analyzer mode, continuum survey with a range of 50–1500 Da and scan time of 0.3 s. For MS/MS a gradient fragmentation energy of 20 V to 30 V was used. When acquired, data were imported in Progenesis (Waters/Non-Linear Dynamics, USA) and processed using default parameters - all runs were assessed for suitability as an alignment reference, with automatic alignment. Peak picking was done at automatic sensitivity (default setting), no retention time limits, fragment sensitivity of 0.2% base peak, and all available adducts. Compounds were annotated using an in-house metabolite database and publicly available fragmentation spectra databases (e.g. MassBank).

### 2.3 Treatment of *Z. mays* with *R. alba* biomass powder

A controlled pot experiment was conducted to examine the effects of leaf and stem powder of *R. alba* L. on maize (Var. Cs220) growth. The experiment took place in late spring 2024 (April–May) at the Department of Botany, Bacha Khan University, Charsadda. Uniform plastic pots (25.0 cm height × 22.0 cm diameter; volume ~10 L were used and each filled with sterilized soil with respective stem and leaf powder (5, 10, and 15 g.kg^−1^ of soil) of *R. alba*, whereas each treatment consisted of three independent replicate pots. The pot was considered the experimental unit. The mean measurements for each pot were recorded before the statistical analysis, and plants were randomly selected from each pot for data collection. The experiment was conducted without the use of any chemical fertilizers, however, the soil composition consisted of sandy loam, exhibiting a pH of 7.64 and an electrical conductivity (EC) of 1.2 dS/m. Nutrient analysis revealed moderate levels of organic matter (1.6%), 58.75 mg.kg^−1^ of nitrogen, 9.50 mg.kg^−1^ of phosphorus, and 110.34 mg.kg^−1^ of potassium. Ten maize seeds were sown at uniform spacing in each pot, which were also uniformly distanced from one another, under semi-controlled greenhouse conditions. Maize seedlings were randomly selected from each pot for the measurement of growth variables, ensuring an accurate representation across the experimental sections. The environment maintained a natural light/dark photoperiod, with average day and nighttime temperatures (28.0 ± 2.2 °C and 18 ± 2.0 °C) and relative humidity 60-70% throughout the experimental period.

The corresponding *R. alba* stem and leaf powders were well mixed separately in the soil (at 5, 10, or 15 g/kg of soil) prior to seed sowing, and the pots were watered every day based on need, while control pots received no powder and were only irrigated with distilled water. After seven days of germination, maize seedlings were randomly selected from each treatment, and plumule and radicle lengths measured using a ruler (in centimeters). No toxicity effect of the R. alba treatment was observed. Following a 30-day growth period, ten maize seedlings were growing in each plastic pot from ten seeds, and essential parameters including root and leaf number was documented manually by counting the visible primary roots and fully expanded leaves on each plant.

### 2.4 Biochemical analyses

#### 2.4.1 Chlorophyll concentration

Chlorophyll a (Chl a), chlorophyll b (Chl b), and carotenoids content in maize leaves was measured following the protocol of Caesar et al. (2018). Fresh, fully expanded leaves were homogenized in 2 mL of 80% acetone, and the total volume was subsequently adjusted to 7 mL. Absorbance measurements were performed using UV spectrophotometer (Shimadzu-China; model UV-1780) at wavelengths of 480 nm, 510 nm, 645 nm, and 663 nm employing the formulas:


$$Chlorophyll\;a.\;(\frac{mg}{g})=\frac{12.3\;D663-0.86\;D645}{d\;x\;1000\;x\;w}$$

$$Chlorophyll\;b.\;(\frac{mg}{g})=\frac{19.3\;D645-0.86\;D663}{d\;x\;1000\;x\;w}$$

Total Chlorophyll=Chl. a+Chl. b

$$Carotenoids=\frac{\left(1000\;A470-1.82\;Chl\;a-85.02\;Chl\;b\right)}{198}$$

#### 2.4.2 Protein concentration

The protein content in the maize samples was analyzed using the Kruger (2009) method. Samples of 1 g were ground in 1 mL of phosphate buffer and then centrifuged at 3000 rpm for 10 minutes. Subsequent to the dilution of 0.1 mL of the supernatant with distilled water to achieve a total volume of 1 mL, 1 mL of Reagent C was incorporated. Following a 10-minute stirring period, 0.1 mL of Reagent D was introduced, and the mixture was allowed to stand for 30 minutes. Absorbance was measured at 650 nm using Folin reagent as a blank.

##### 2.4.2.1 Preparation of reagents

Reagent A consists of 2 g of Na_2_CO_3_, 0.4 g of NaOH, and 1 g of sodium-potassium tartrate, dissolved in 100 mL of distilled water.

Reagent B is formulated by dissolving 0.5 g of CuSO_4_·5H_2_O in 100 mL of distilled water.

Reagent C consists of 50 mL of Reagent A and 1 mL of Reagent B.

Reagent D consists of a 1:1 mixture of Folin-phenol reagent and distilled water.

#### 2.4.3 Proline concentration

The evaluation of proline concentrations in maize leaves was conducted using the methodology established by Ábrahám et al. (2010). Leaf samples (0.2 g) were homogenized in 5 mL of 3% sulphosalicylic acid and incubated at 5 °C for 24 hours. The solution was subjected to centrifugation at 4000 rpm for a duration of 5 min. Two milliliters of the supernatant were combined with 2 mL of acid ninhydrin reagent, formulated from 6M phosphoric acid, glacial acetic acid, and 1.25 g of ninhydrin. The mixture underwent heating in a water bath for 60 min., subsequently cooled, and extracted using 4 mL of toluene. The toluene layer was isolated, and its absorbance was quantified at 520 nm. Toluene functioned as a control sample.

#### 2.4.4 Total sugar content

The total sugar content was assessed following the protocol of Buysse and Merckx (1993) by utilizing the phenol-sulphuric acid method. Homogenization of 0.5 g of fresh maize leaf tissue was performed in an 80% methanol solution. The extract (0.5 mL) was diluted to a final volume of 10 mL using distilled water and subsequently centrifuged at 3000 rpm for a duration of 10 minutes. 0.5 mL of the supernatant was combined with 1 mL of 80% phenol and incubated for 10 minutes. Following this, 5 mL of concentrated sulphuric acid was introduced, and the solution was incubated for a duration of 4 hours. Absorbance was measured at 420 nm.

### 2.5 Statistical analysis

The replicated data were analyzed utilizing a one-way analysis of variance (ANOVA) via SPSS 16.0 software. We assessed the data for normality and homogeneity of variance using the Shapiro–Wilk Levene’s test before running ANOVA, respectively. In cases where significant differences were identified, pairwise comparisons were conducted using Duncan’s multiple range test (DMRT) at a significance level of p=0.05 to ascertain which pairs of means exhibited significant differences. ResultsResults are presented as mean ± standard error (SE). Bar graphs were generated using the “ggplot2” R package (version 3.4.2), and Principal Component Analysis (PCA) was conducted using the “FactoMineR” R package (version 2.6) with visualization via “factoextra” (version 1.0.7).

## 3 Results

### 3.1 Biochemical composition of *Rosa alba* leaf and stem plant material

Using dedicated GC-MS and UHPLC-MS protocols, we identified 106 metabolite peaks in total (**Tables 1**, **2**). More detailed information about these metabolites, including the data from the four biological replicates per each tissue, are given in **Supplementary Table 1** (metabolites identified by GC-MS) and **Supplementary Table 2** (metabolites identified by UHPLC-MS). Besides the most abundant primary metabolites such as the most common sugars, amino acids, and organic acids, we identified a number of vitamins (pantothenic acid), plant hormones (trans-zeatin-O-glucoside), and many secondary metabolites such as gallic acid, 3,4-Dihydroxybenzoic acid, quinic acid, suberic acid, myricitrin, caffeic acid, cinnamic acid, isorhamnetin-3-O-rutinoside, kaempferol-3/7-O-glucoside, isorhamnetin-3-O-glucoside, and kaempferol-3-O-rutinoside (**Tables 1**, **2**).

**Table 1:** Metabolites quantified by GC-MS in aqueous *Rose alba* extracts.

Metabolite	Annota-tion level	m/z	RT	Leaf mean peak area	Stem mean peak area	BH-p-value	Signifi-cance
Glycolic acid	1	177	3.56	38024241.1	20978142.7	0.03266	*
Alanine	1	116	3.62	523766778	695843955	0.106665	ns
Valine	1	144	4.68	176329502	156985863	0.412054	ns
Ethanolamine	2	174	4.87	115253277	171230311	0.061318	ns
Glycerol	1	205	5.04	434407364	324997042	0.046272	*
Leucine	1	158	5.18	148623150	73759927.4	0.014058	*
Isoleucine	1	158	5.41	109819546	67204321.7	0.027272	*
Glycine	1	174	5.55	38166771.7	46521279.9	0.239082	ns
Urea	1	189	5.68	35409802	34220590.7	0.882279	ns
Phosphoric acid	1	299	5.7	1235720881	1454215025	0.360358	ns
Proline	1	142	5.74	210764609	1127456383	0.03266	*
Benzoic acid	1	105	5.8	29691152.5	36898038.4	0.515362	ns
Glyceric acid	2	292	5.89	109829990	43704014.9	0.013668	*
Serine	1	204	6.1	42455560	125608633	0.03227	*
Succinic acid	1	247	6.13	185375150	41827724.1	0.01278	*
Fumaric acid	1	245	6.14	87914799.9	119354960	0.106665	ns
Threonine	1	219	6.26	25778006.9	49887752.7	0.031688	*
Maleic acid	1	245	6.29	19130003.6	7041493.73	0.013806	*
Pipecolic acid	3	156	6.32	7125249.93	34859596	0.01278	*
Nicotinic acid	1	180	6.36	13722581.2	13520763.1	0.89	ns
Erythritol	1	217	7.02	39913167.4	22410630.9	0.017135	*
Malic acid	1	233	7.44	857467247	868374304	0.89	ns
4-hydroxy-Proline	1	230	7.54	8229293.3	16109818.9	0.040571	*
4-Aminobutanoic acid	1	174	7.57	784616800	1172168196	0.061318	ns
Aspartic acid	1	232	7.68	48495643.5	218194276	0.032626	*
Threonic acid	1	292	7.81	231206233	70243116.9	0.01278	*
Salicylic acid	1	267	8.06	28420775.9	35976584.6	0.213	ns
Asparagine	2	188	8.13	851010.503	64996487.9	0.078494	ns
Pyroglutamic acid	1	156	8.42	87409872.4	319246899	0.01562	*
Glutamic acid	1	246	8.48	131773767	68101377.1	0.022276	*
Xylose 2	1	307	8.5	77261893.1	47901821	0.037138	*
Rhamnose	1	117	8.81	191823847	41634441.4	0.01278	*
Phenylalanine	1	192	8.84	42620168.1	30479814.2	0.1136	ns
Fucose SP	1	117	8.99	20347922.8	15830708.6	0.137987	ns
Ornithine 2	1	142	9.55	11620536.2	100827072	0.040571	*
Shikimic acid	1	204	9.82	1839984036	1245055467	0.014329	*
Quinic acid	1	345	9.83	3887465021	4597170647	0.655567	ns
Fructose 1	1	217	9.85	3379207921	3319518632	0.805794	ns
Glutamine	1	156	9.91	19905905.1	80414616.2	0.057945	ns
Citric acid	1	273	9.93	1077492901	1899022265	0.03266	*
Fructose 2	1	217	9.95	3316866541	2573121361	0.031688	*
Galactose MP	1	160	9.97	127105113	120876615	0.63178	ns
Glucose 1	1	160	10.04	2158637714	1856855184	0.080467	ns
Glucose 2	1	160	10.15	913348961	772655587	0.080467	ns
Lysine	1	156	10.24	20086910.2	21064220.6	0.841077	ns
Galactonic acid gamma lactone	1	189	10.31	287163469	378318825	0.104249	ns
2-ketoglutaric acid	3	173	10.44	17214578.3	16433898.6	0.858358	ns
Galactonic acid	3	333	10.65	194078085	176609736	0.670426	ns
Tyrosine 218 mz	1	218	10.86	96032220.3	295942245	0.048043	*
Tyrosine 280 mz	1	280	10.86	14182897.5	26848029.2	0.09443	ns
Gallic acid	2	458	10.98	229933442	144427921	0.06816	ns
Myo-inositol	1	305	11.09	3401593883	2209732756	0.01278	*
4-Coumaric acid	1	293	11.18	63391830.2	80821983.1	0.370778	ns
linamarin related	3	204	11.75	19535364.2	8831924.92	0.01278	*
Glyceryl-Glycoside	3	204	12.57	375633224	107726081	0.01562	*
Tryptophan	1	202	13.03	11686682.4	122830228	0.080467	ns
disaccharide 1	3	204	13.4	125825824	6721700.6	0.01562	*
disaccharide 2	3	204	14.09	185144027	36334504.4	0.208468	ns
Sucrose	1	361	14.23	123328262	2514052597	0.01278	*
Maltose MP	1	204	14.71	23643464	25661100.7	0.828703	ns
Cellobiose?	3	361	15.17	42998393.5	30212024.6	0.371782	ns
beta-Gentiobiose	3	361	15.28	28327030	24423887.8	0.789016	ns
Terpene glycoside	3	297	15.69	67541715.3	45645955.7	0.27122	ns
Galactinol	1	204	15.76	823974418	571610741	0.338294	ns
Unknown CGA derivative	3	345	15.94	268859314	291128033	0.858358	ns
Unknown 297 mz	3	297	16	64003762.5	61251474.1	0.89	ns
Catechine	2	368	16.19	58869447.2	109799728	0.355	ns
Unknown disaccharide 3	3	204	16.43	871046200	193632410	0.062021	ns
Unknown disaccharide 4	3	361	17.39	36988240.7	148341773	0.109889	ns

The asterisk (*) indicates statistically significant differences, *p* ≤ 0.05.

**Table 2:** Metabolites quantified by UHPLC-MS in aqueous *Rose alba* extracts.

Metabolite	Anno-tation level	m/z	RT	Leaf mean abundance	Stem mean abundance	q-value	Signifi-cance
Ornithine	2	131.0827	0.59	128.8151	860.4740	0.0000	****
Gluconic acid	2	195.0516	0.66	25306.5177	22770.0713	0.0227	*
Saccharic acid	2	209.0303	0.65	4061.2315	2166.8790	0.0001	***
3-Deoxy-D-manno-2-octulosonic acid	2	237.0617	0.66	7472.4692	4483.9903	0.0083	**
Uridine-5’-diphosphogalactose	2	565.0454	0.69	3467.1349	2190.3725	0.0023	**
Malic acid	2	133.0144	0.89	7136.3183	5118.4865	0.0505	ns
Uric acid	2	167.0210	1.36	72.1525	487.2995	0.0000	****
Citric acid	2	191.0199	1.34	39310.0703	53141.4296	0.0020	**
Pyroglutamic acid	2	128.0354	1.51	71.1863	272.7527	0.0001	****
Uridine	2	243.0623	1.95	2032.9312	1867.0712	0.0267	*
3-Hydroxy-3-Methylglutaric acid	2	161.0454	2.31	136.6784	65.8745	0.0012	**
Guanosine	2	282.0841	2.77	513.7697	459.8781	0.0237	*
Pantothenic acid (Vitamin B5)	2	218.1037	3.67	116.9156	81.5031	0.0888	ns
trans-zeatin-O-glucoside	2	380.1563	3.87	206.9286	55.8978	0.0003	***
3,4-Dihydroxybenzoic acid	2	153.0193	3.88	965.5586	415.2864	0.0010	***
Succinoadenosine	2	382.0998	3.82	218.2519	108.3681	0.0010	***
Uridine diphosphate-N-acetylglucosamine	2	606.0738	0.71	1465.2265	900.1574	0.0009	***
Arginine	1	173.1044	0.59	502.6745	3247.0657	0.0000	****
D-()-Raffinose	1	503.1612	0.67	6670.1180	3725.3102	0.0014	**
Fructose 6-phosphate	1	259.0224	0.61	1535.3712	1395.6420	0.0009	***
Quinic acid	1	191.0561	0.75	149247.4790	109006.4143	0.0515	ns
L-Galactono-1,4-lactone	1	177.0405	0.66	967.5835	750.6056	0.1537	ns
Monosaccharide	1	179.0561	0.66	8478.7911	6235.8893	0.0811	ns
Disaccharide	1	341.1082	0.78	3939.0555	49348.7123	0.0000	****
Shikimic acid	1	173.0452	0.74	1356.4288	811.4460	0.0006	***
Suberic acid	2	173.0818	6.73	2034.0305	1826.3610	0.0011	**
Myricitrin	2	463.0876	7.24	10946.0416	995.2495	0.0000	****
Caffeic acid	1	179.0349	5.56	119.1463	11.0350	0.0002	***
Cinnamic acid, 4-hydroxy-, trans-	1	163.0400	6.65	208.1135	216.4185	0.0019	**
Q3G6R	2	609.1457	6.37	307.8748	1729.3675	0.0001	****
4CGA	2	353.0877	5.26	373.3799	78.3408	0.0002	***
Isorhamnetin-3-O-rutinoside	2	623.1609	7.69	7632.6745	664.5976	0.0001	****
Kaempferol-3/7-O-Glucoside	2	447.0923	7.88	286839.2798	15560.2595	0.0000	****
Isorhamnetin-3-O-glucoside	2	477.1031	7.96	530.6113	102.4423	0.0002	***
Kaempferol-3-O-rutinoside	2	593.1504	7.55	6841.1654	369.0943	0.0000	****

The asterisks **, ***, **** indicate statistically significant differences of *p* ≤ 0.01, *p* ≤ 0.001, and *p *≤ 0.0001, respectively.

While some of these metabolites were equally abundant in the leaves and the stems, others such as trans-zeatin-O-glucoside, 3,4-Dihydroxybenzoic acid, myricitrin, caffeic acid, isorhamnetin-3-O-rutinoside, kaempferol-3/7-O-glucoside, isorhamnetin-3-O-glucoside, and kaempferol-3-O-rutinoside were substantially more abundant in the leaves (**Table 2**). Several amino acids, such as proline, serine, aspartic acid, and asparagine, as well as a few other metabolites such as uric acid and pyroglutamic acid were much more abundant in the stems (**Tables 1**, **2**).

### 3.2 Morphological analysis of the application of leaf and stem powdered biomass of *R. alba* on *Z. mays*

**Figure 1:**
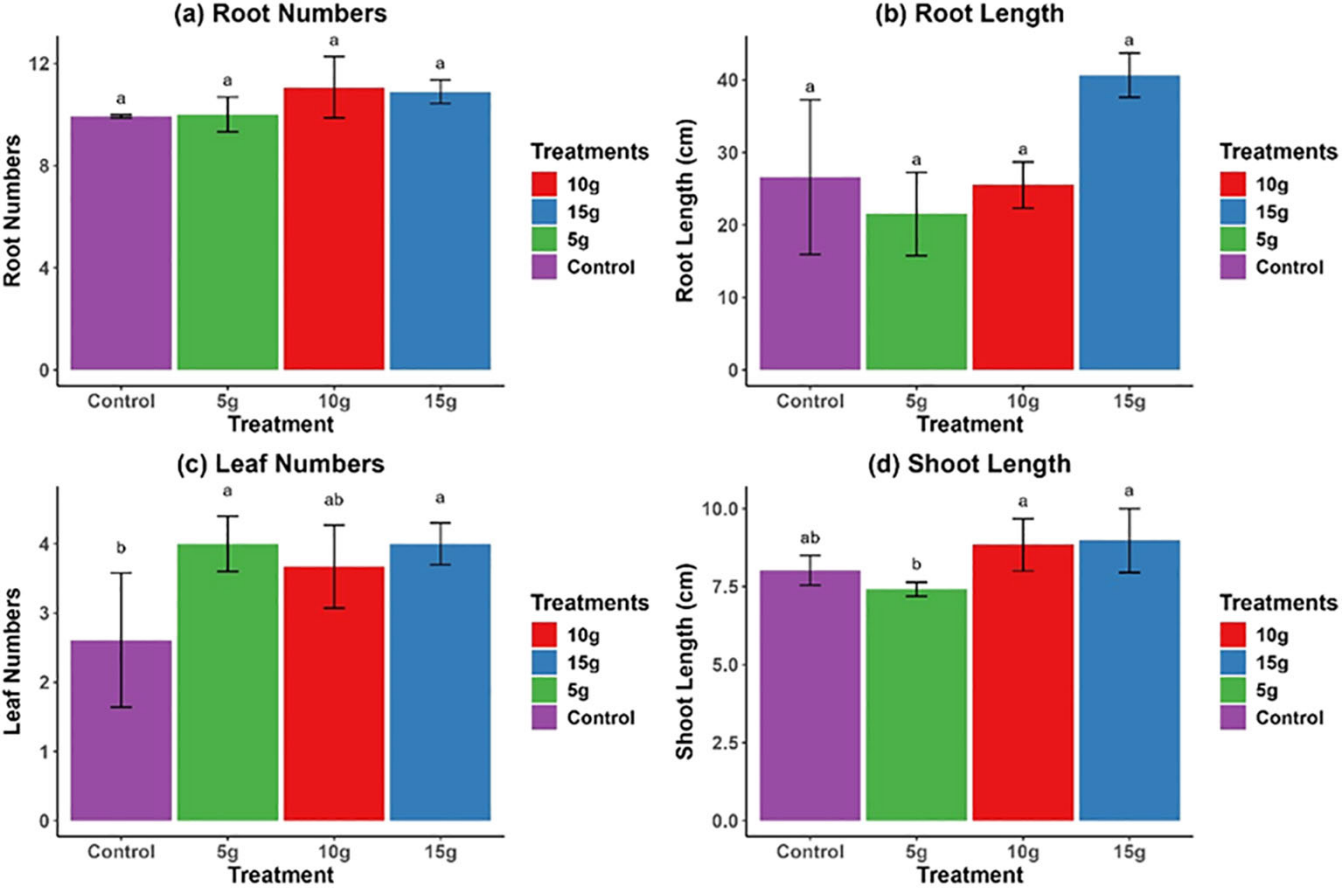
Impact of 5 g, 10 g, and 15 g leaf powdered biomass of *R. alba* L. on *Z. mays* L. root numbers **(a)**, root length **(b)**, leaf numbers **(c)**, and shoot length **(d)**. The 5 g, 10 g, and 15g powdered biomass correspond to 5 g.kg^−1^, 10 g.kg^−1^, and 15 g.kg^−1^ of soil, respectively. The data are expressed as the mean ± standard error (SE) of three biological replicates, each replicate consisting of 10 different plants. Lowercase letters positioned above the bars signify statistically significant differences (p < 0.05) as determined by Duncan’s Multiple Range Test (DMRT).

**Figure 2:**
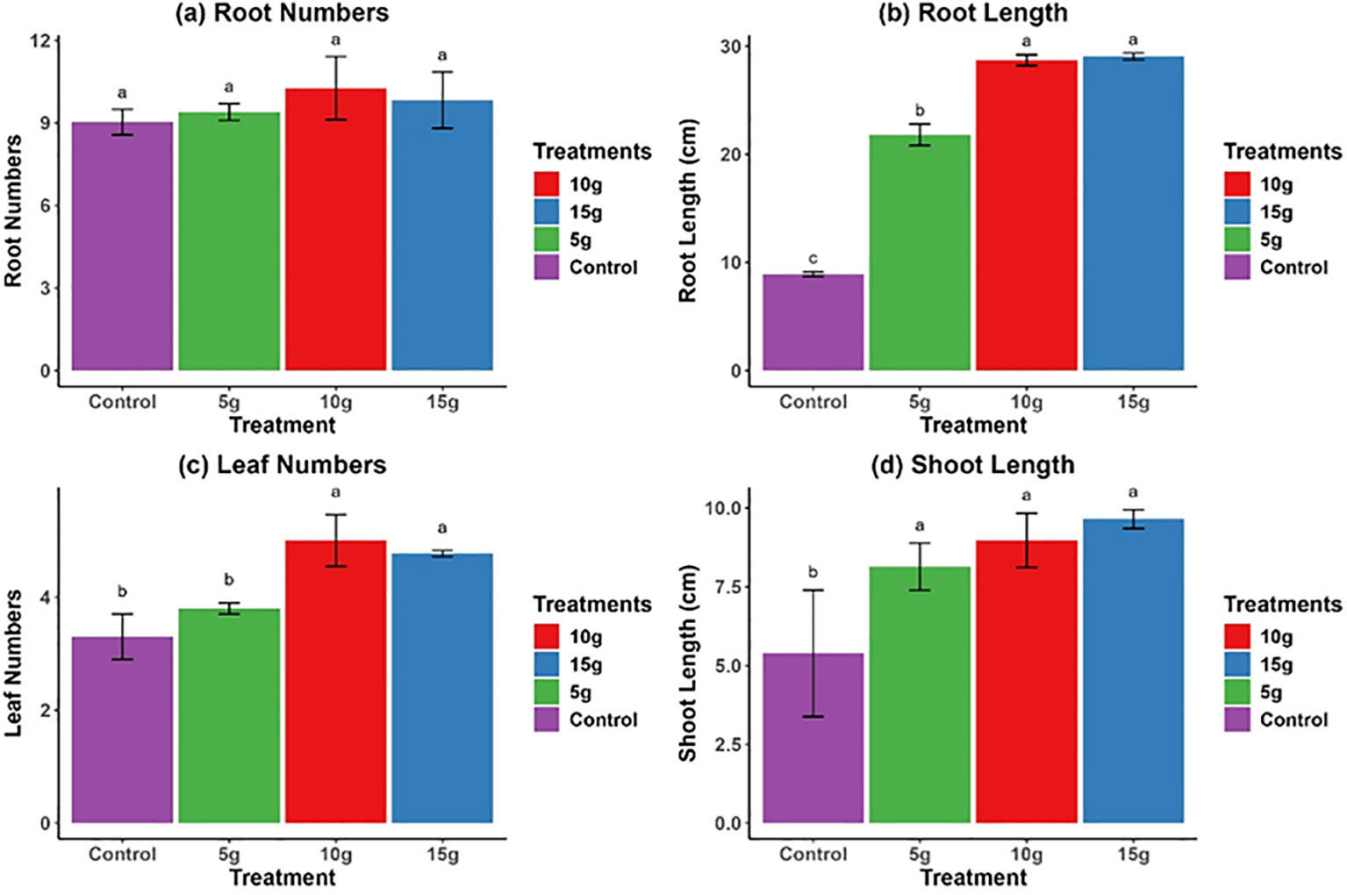
Impact of 5 g, 10 g, and 15 g stem powdered biomass of *R. alba* L. on *Z. mays* L. root numbers **(a)**, root length **(b)**, leaf numbers **(c)**, and shoot length **(d)**. The data are expressed as the mean ± standard error (SE) of three biological replicates, each replicate consisting of 10 different plants. Lowercase letters positioned above the bars signify statistically significant differences (p < 0.05) as determined by Duncan’s Multiple Range Test (DMRT).

While the application of *R. alba* L. leaf powdered biomass had little or no effect on the growth parameters of *Z. mays* seedlings (**Figure 1**), the stem powdered biomass of *R. alba* L. exhibited a significant positive effect on maize growth parameters, especially on the leaves number, leaf length, and root length (**Figure 2**). Results of root number showed no significant effect (**Figure 2a**), whereas root length showed the greatest improvement at the 15 g dose (30.0 ± 3.15* cm) (**Figure 2b**). Leaf development exhibited a similar trend, with the 10 g treatment yielding the highest leaf count (4.0 ± 0.40*) (**Figure 2c**), and shoot length also reaching its maximum under this treatment (9.0 ± 0.30* cm) (**Figure 2d**). The findings indicate that both 10 and 15 g doses of *R. alba* L. stem powdered biomass significantly (*p* < 0.05) enhance root and shoot development, underscoring their potential as natural growth stimulants.

### 3.3 Biochemical analysis of leaf and stem of maize treated with *R. alba*

#### 3.3.1 Effect of *R. alba* L. leaf and stem powdered biomass on photosynthetic pigments of *Z. mays* L.

**Figure 3:**
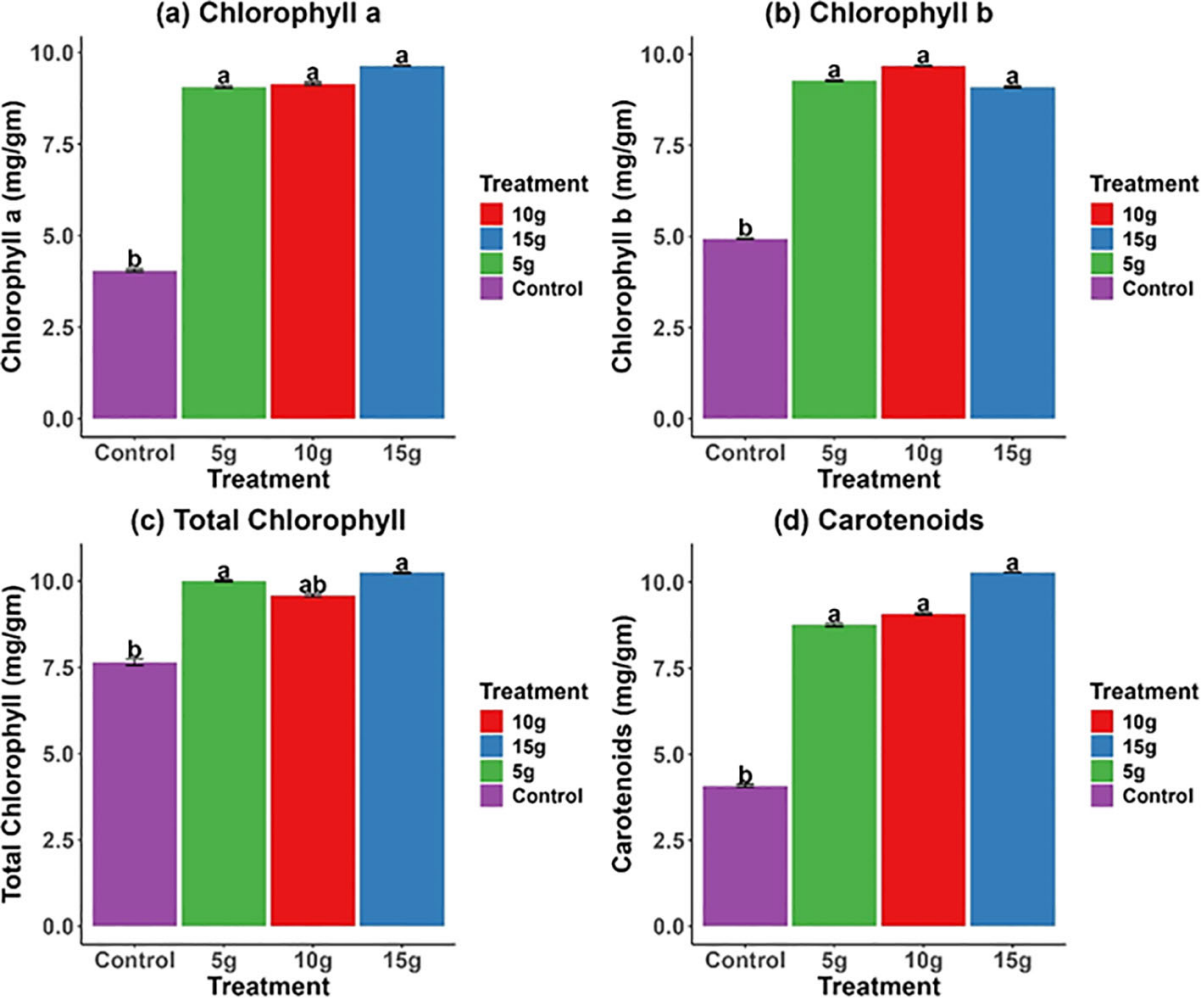
The impact of *R. alba* L. leaf powdered biomass on the photosynthetic pigments of *Z. mays* L. includes: **(a)** Chlorophyll a, **(b)** Chlorophyll b, **(c)** Total Chlorophyll, and **(d)** Carotenoids. The data are expressed as the mean ± standard error (SE) of three biological replicates, each replicate consisting of 10 different plants. Lowercase letters positioned above the bars signify statistically significant differences (p < 0.05) as determined by Duncan’s Multiple Range Test (DMRT).

The use of *R. alba* L. leaf powdered biomass markedly enhanced the levels of pigments produced by photosynthesis in *Z. mays* plants (**Figure 3**). Chlorophyll a content was significantly elevated in all treatments relative to the control, with the 15 g treatment exhibiting the highest value (10.0 ± 0.5* mg.g^−1^) (**Figure 3a**). Chlorophyll b content exhibited a notable increase in response to the 10 g and 15 g treatments (**Figure 3b**). The total chlorophyll content exhibited a significant enhancement, with peak levels recorded at 15 g (**Figure 3c**). Carotenoid levels reflected these trends, with the 15g treatment attaining the highest concentration (10.0 ± 0.4* mg.g^−1^) (**Figure 3d**). The findings demonstrate that *R. alba* L. leaf powdered biomass, especially at elevated doses, promote the accumulation of photosynthetic pigments, thereby enhancing photosynthetic efficiency and overall plant productivity.

**Figure 4:**
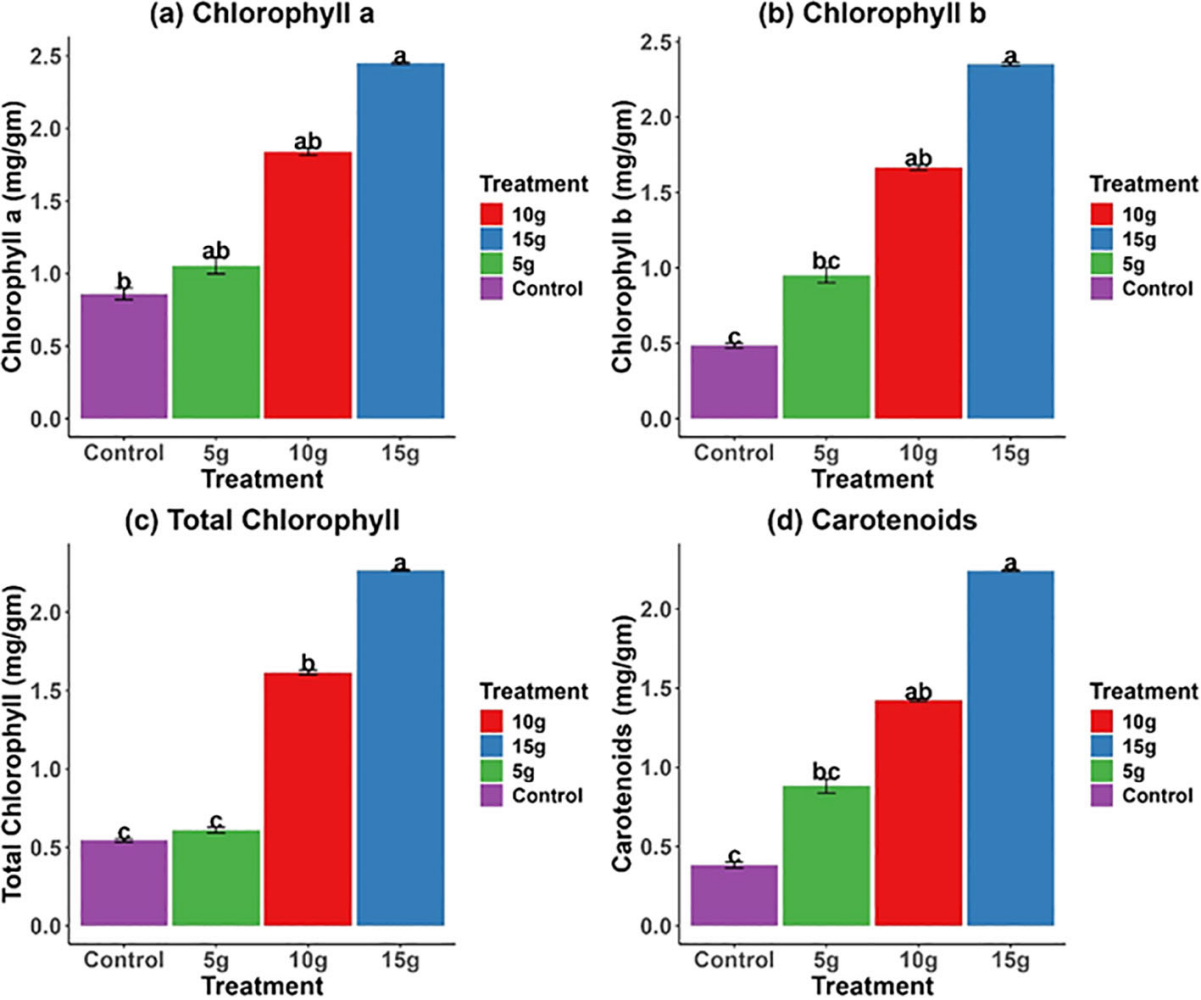
Effect of *R. alba* L. stem powdered biomass on photosynthetic pigments of *Z. mays* L. **(a)**: Chlorophyll a **(b)**: Chlorophyll b **(c)**: Total Chlorophyll **(d)**: Carotenoids. The data are expressed as the mean ± standard error (SE) of three biological replicates, each replicate consisting of 10 different plants. Lowercase letters positioned above the bars signify statistically significant differences (p < 0.05) as determined by Duncan’s Multiple Range Test (DMRT).

The findings indicated a notable enhancement in photosynthetic pigment levels after the application of *R. alba* L. stem powdered biomass (**Figure 4**). Chlorophyll a concentrations were maximal in the 15g treatment (2.5 ± 0.1* mg/g), with the 10g treatment exhibiting slightly lower levels (**Figure 4a**). Chlorophyll b and total chlorophyll content displayed comparable trends, with the 15g treatment reaching the highest levels (**Figures 4b, c**). The carotenoid content exhibited a significant increase with higher powdered biomass concentrations, with the 15g treatment yielding the highest values (2.0 ± 0.1* mg.g^−1^) (**Figure 4d**). The findings indicate that *R. alba* L. stem powdered biomass positively influence the production of photosynthetic pigments, thereby enhancing the photosynthetic capacity of the plants.

#### 3.3.2 Analysis of protein and sugar concentrations

**Figure 5:**
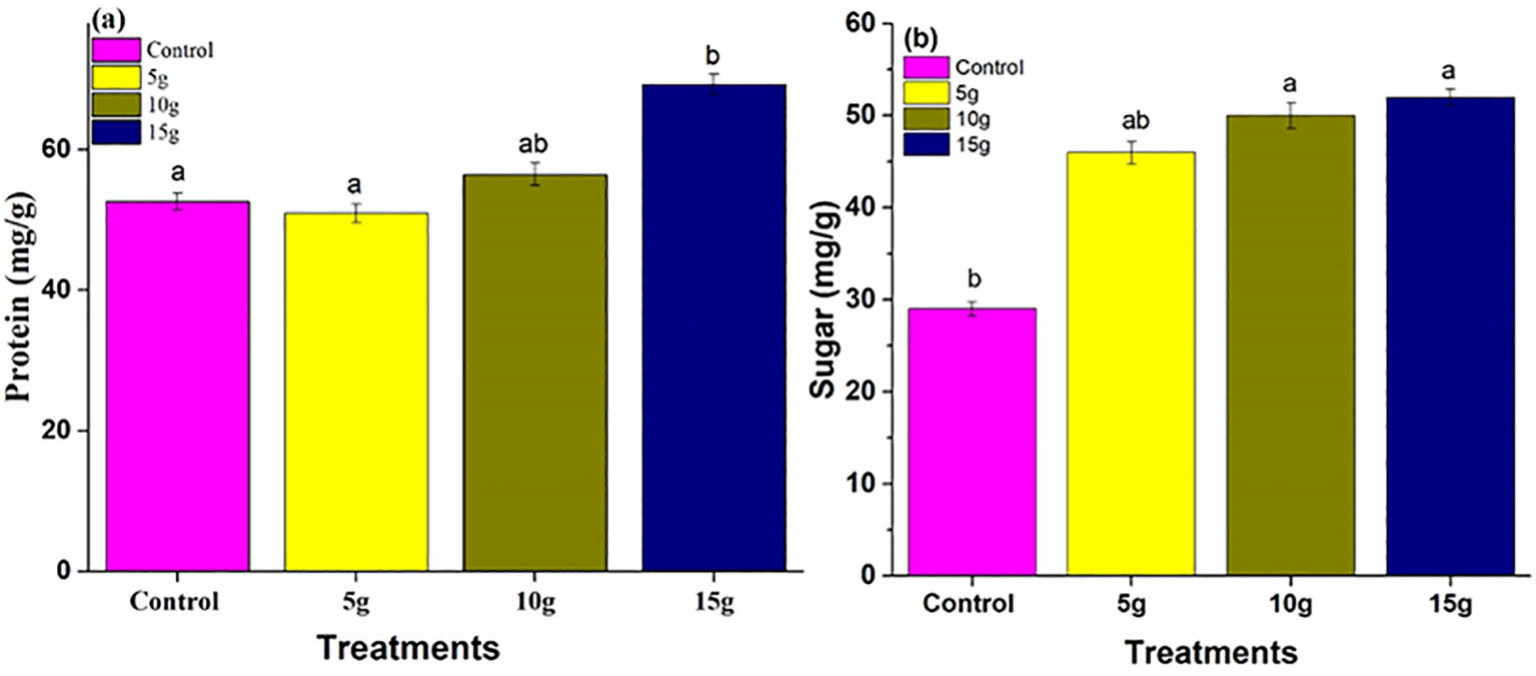
Impact of *R. alba* L. leaf powdered biomass on protein **(a)** and sugar **(b)** levels. The data are expressed as the mean ± standard error (SE) of three biological replicates, each replicate consisting of 10 different plants. Lowercase letters positioned above the bars signify statistically significant differences (p < 0.05) as determined by Duncan’s Multiple Range Test (DMRT).

The application of *R. alba* L. leaf powdered biomass notably affected the protein concentrations in maize plants (**Figure 5**). The 15 g treatment produced the highest protein content (65 ± 2.0* mg.g^−1^), followed by the 10 g treatment (**Figure 5a**), both demonstrating a relatively similar to the control group. The findings suggest that higher concentrations of *Rosa alba* L. leaf powdered biomass may enhance metabolic activity, leading to increased protein synthesis and sugar accumulation (**Figure 5b**), thereby contributing to improved plant growth and vigor.

**Figure 6:**
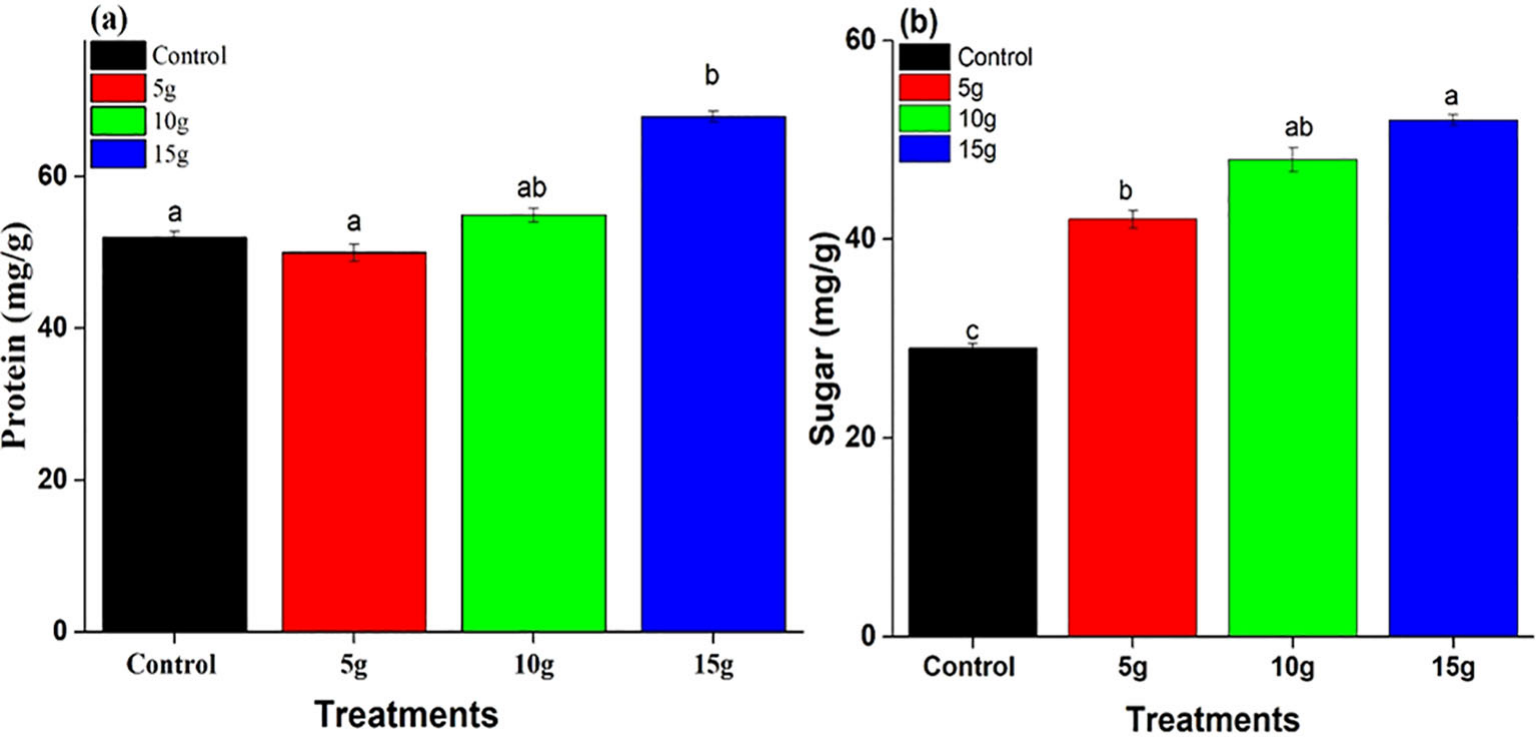
Effect of *Rosa alba* L. stem powdered biomass on **(a)** protein and **(b)** sugar contents. The data are presented as mean ± standard error (SE) of three replicates. Different lowercase letters above the bars indicate statistically significant differences among treatments at p < 0.05 according to Duncan’s Multiple Range Test (DMRT).

The biochemical analysis indicated a notable increase in protein and sugar levels following treatments with *R. alba* L. stem powdered biomass (**Figure 6**). The 15 g treatment resulted in the highest protein content (65 ± 2.0* mg.g^−1^) (**Figure 6a**), with sugar content also reaching its maximum at this dosage (45 ± 1.5* mg.g^−1^) (**Figure 6b**). Both the 5 g and 10 g treatments demonstrated moderate enhancements relative to the control group. The findings indicate that *R. alba* L. stem powdered biomass, especially at a concentration of 15g, promote the synthesis and accumulation of proteins and sugars, thereby improving nutrient assimilation and plant growth.

#### 3.3.3 Effect of *R. alba* L. leaf and stem powdered biomass on proline contents

**Figure 7:**
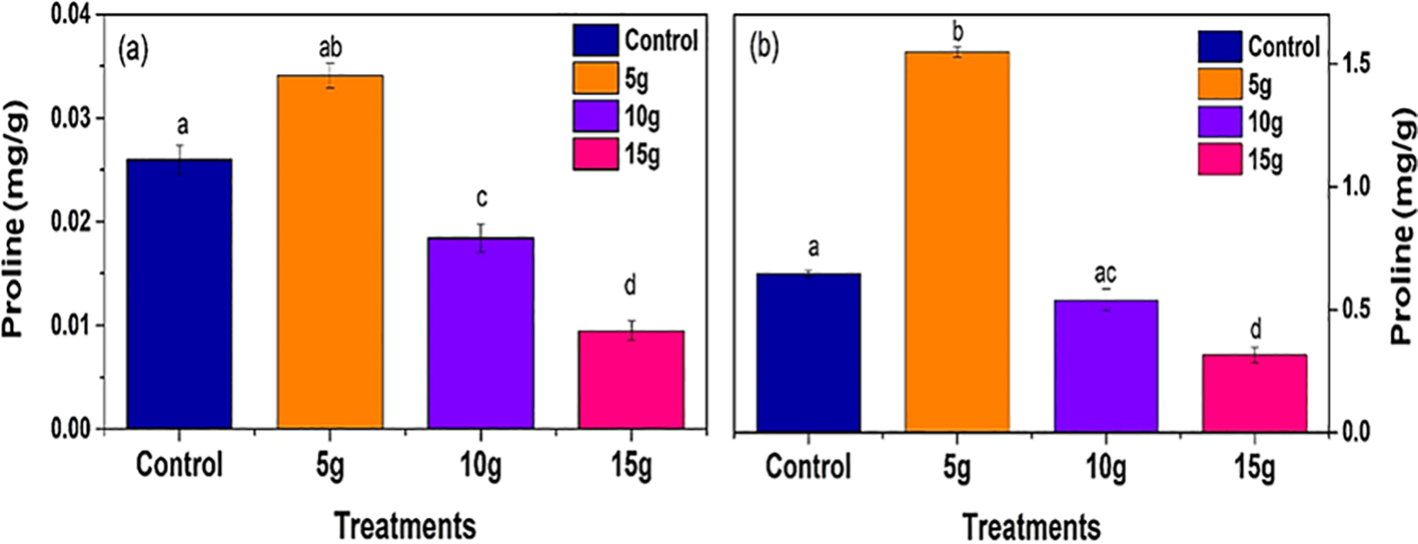
Impact of *R. alba* L. leaf **(a)** and stem **(b)** powdered biomass on proline contents. The data are expressed as the mean ± standard error (SE) of three biological replicates, each replicate consisting of 10 different plants. Lowercase letters positioned above the bars signify statistically significant differences (p < 0.05) as determined by Duncan’s Multiple Range Test (DMRT).

The proline content, which serves as an indicator of plant stress response, exhibited a significant decrease with higher concentrations of *R. alba* L. leaf powdered biomass (**Figure 7a**). The 5g treatment exhibited a moderate decrease in proline content, whereas the 10 g and 15 g treatments produced the lowest proline levels (0.01 ± 0.001* mg.g^−1^) compared to control. The findings indicate that increased doses of *R. alba* L. leaf powdered biomass mitigate stress conditions in maize plants, presumably by enhancing physiological stability and diminishing stress-related responses.

Proline levels, commonly linked to plant stress responses, exhibited a progressive decline with escalating doses of *R. alba* L. stem powdered biomass (**Figure 7b**). The 5g treatment demonstrated the highest proline content (1.5 ± 0.1* mg.g^−1^), whereas the 15 g treatment showed the lowest levels (0.5 ± 0.05* mg.g^−1^). The reduction in proline content with increasing powdered biomass concentration suggests improved physiological conditions and reduced stress in treated plants, likely due to enhanced nutrient availability and metabolic balance.

### 3.4 PCA analysis and structural model

**Figure 8:**
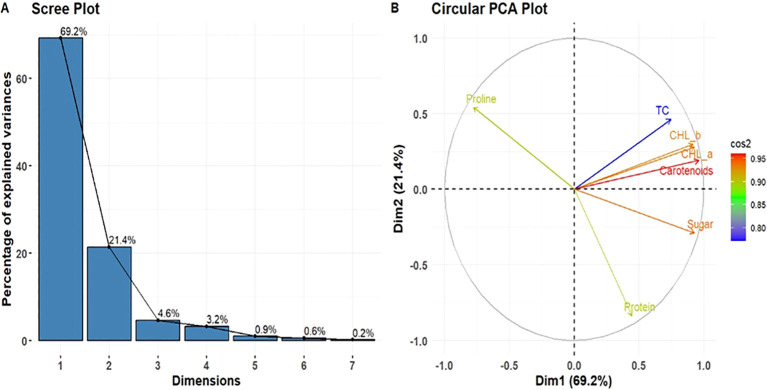
Analysis of principal components derived from measured variables. **(A)**, Scree plot; **(B)**, circular PCA plot illustrating the contribution of variables to Dimension 1 (21.4%) and Dimension 2 (10.9%).

**Figure 9:**
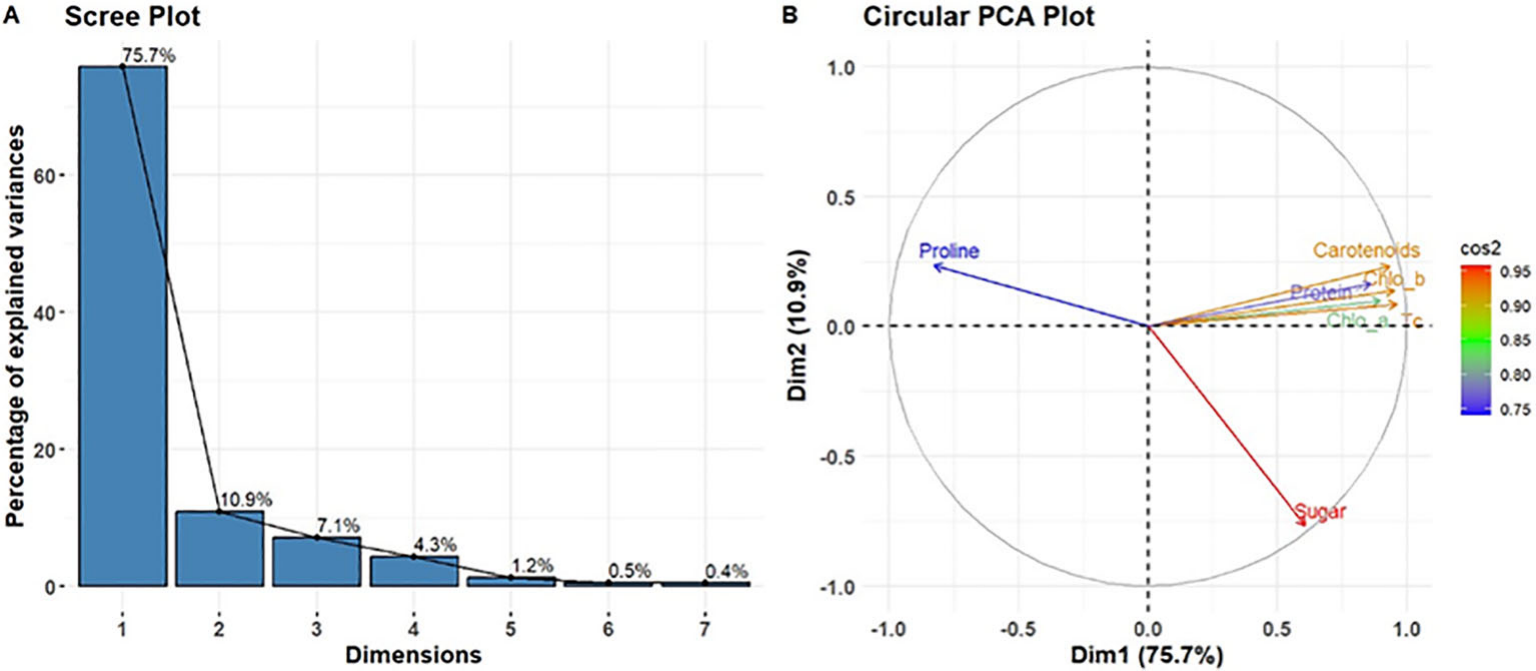
PCA of biochemical parameters in maize under different treatments. **(A)**, Scree plot; **(B)**, circular PCA plot illustrating the contributions of variables to Dimension 1 (75.7%) and Dimension 2 (10.9%). .

The PCA of the leaf powdered biomass (**Figure 8**) revealed that PC1 and PC2 accounted for 69.2% and 21.4% of the total variation, respectively. PC1 had a strong correlation with features such as photosynthetic pigments (chl “a”, “b” total chl. and carotenoids) and sugar. PC2 had a stronger association with proline and protein. In the stem powdered biomass (**Figure 9**), PC1 accounted for a substantial portion of the variance (75.7%), whereas PC2 accounted for about 10.9%. In this context, sugar exhibited a significant negative loading on PC1, but proline was prominent on PC2. Chl “a”, “b”, total chl, and carotenoids were clustered at PC1. These PCA results indicate that leaf and stem powdered biomass exhibit distinct biochemical response patterns. The PCA biplots clearly indicated that treatments clustered along PC1, as demonstrated by the sample scores and variable loadings. In the stem-based PCA, soluble sugar exhibited a negative loading on PC1, while photosynthetic pigments (chlorophyll a, b, total chlorophyll, and carotenoids) and protein content demonstrated significant positive loadings. PC2 exhibited a strong association with proline. The score graphs indicated that the treatment induced biochemical changes, as evidenced by the differences between the treated and control samples. The impacts of leaf powdered biomass are more uniform across characteristics, whereas the effects of stem powdered biomass exhibit more variability, particularly in sugar and proline concentrations.

**Figure 10:**
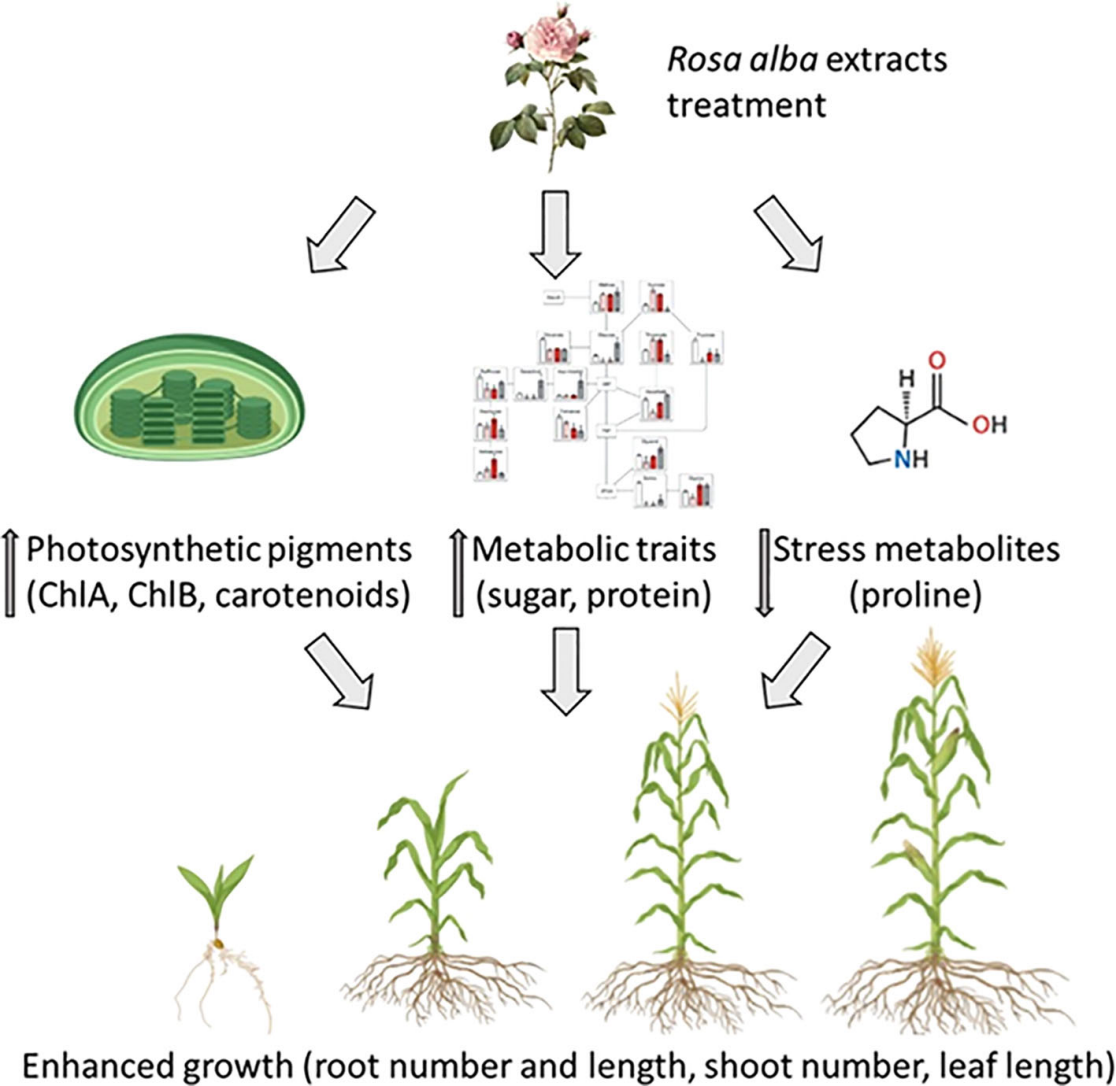
Structural Equation Model illustrating the effects of *R. alba* L. treatments (5, 10, 15g. kg^-1^ of soil) on the growth of *Z. mays* via increase in photosynthetic pigments, metabolic traits (sugars, proline), and reduction of stress (decrease in proline). The figure was prepared using biorender.com.

**Figure 10** illustrates the impact of varying concentrations of *R. alba* leaf and stem powdered biomass (5, 10, and 15g/kg of soil) on the overall growth of maize. The structural model indicates that the treatments accelerated development by increasing the levels of photosynthetic pigments (chl “a”, “b”, total chl. and carotenoids), improving metabolic characteristics (sugar and protein), and altering the levels of stress-related metabolites (proline). The modifications in the plant physiological functions collectively enhanced critical growth factors, including root and shoot length, leaf number, and overall plant health.

## 4 Discussion

This research demonstrates the potential of *R. alba* L. powdered biomass to act as growth promoting agent for maize, enhancing leaf numbers, shoot length, and root length. Interestingly, the stem biomass ad much more pronounced activity than the leaf biomass. It may be speculated that differences in metabolic compositions between leaves and stems are responsible for the different biological effects. The complex biochemical compositions of *R. alba* leaves and stems, identified by the comprehensive metabolome analyses, revealed a number of bioactive compounds (hormones, vitamins, essential amino acids, and variety of secondary metabolites) that could contribute to the growth promoting biostimulant effect of *R. alba*. Prior research indicates that *R. alba* encompass a range of secondary metabolites, such as phenolic acids, flavonoids, tannins, and essential oils (Da Silva et al., 2014), which may act as a growth promoters. These compounds are associated with antimicrobial activity, enhanced nutrient uptake, and improved root development (Campobenedetto et al., 2021; Kisiriko et al., 2021; Yasa et al., 2009). Furthermore, the powdered leaves and stems can provide a significant source of organic matter and macronutrients, such as nitrogen, phosphorus, and potassium, thereby enhancing soil fertility and promoting microbial activity (Schenck zu Schweinsberg-Mickan and Müller, 2009), which may enhance nutrient availability and serve as a mild growth stimulant, similar to the mechanisms associated with green manure or compost-based amendments (Bhardwaj et al., 2014). Amino acids in particular, especially abundant in the stem of *R. alba*, can serve as such growth promoting nutrients. The 10 g treatment proved to be particularly effective, promoting both shoot and root development, whereas the 15 g treatment primarily enhanced root elongation. Root development, essential for the acquisition of water and nutrients, indicates enhanced biological processes and utilization of resources following treatment application (Chapman et al., 2012). Excessive doses may induce imbalances that restrict growth enhancement, as evidenced by the minimal improvement observed with the 5 g treatment (Vitousek et al., 2009). These observations align with research highlighting the significance of dose optimization to prevent nutrient stress or toxicity (Eftekharzadeh et al., 2018; Gopalakrishnan et al., 2018; Rouphael and Colla, 2020). The substantial increases in carotenoid, total chlorophyll, chlorophyll a, and chlorophyll b concentrations under the 10 g and 15 g treatments highlight how *R. alba* L. powdered biomass improve photosynthetic efficiency. These tissues’ differential metabolite profiles may be connected to the different effects of leaf and stems powder on growth of maize and pigment content. While stems have a greater amount of amino acids and development-related metabolites, which probably explains their greater impact on early development metrics, leaves have larger quantities of flavonoid and pigments, which alter chlorophyll levels. Chlorophyll pigments play a crucial role in light absorption and energy conversion in photosynthesis, whereas increased levels of these pigments indicate enhanced capacity for photosynthesis and nutrient assimilation (Simkin et al., 2022). Carotenoids contribute to light harvesting and serve as antioxidant agents by scavenging reactive oxygen compounds (ROS), thus mitigating oxidative stress (Ashraf and Foolad, 2007; du Jardin, 2020; Eftekharzadeh et al., 2018; Gopalakrishnan et al., 2018; Hare et al., 1998; Van Oosten et al., 2017). The 15 g treatment, which optimized pigment content, indicates improved photoprotective mechanisms and photosynthetic efficiency, essential for plant productivity under optimal conditions. The lower pigment levels in the control group indicate a lack of external stimuli necessary for the optimization of photosynthetic processes. The results corroborate previous findings that nutrient amendments and bioactive compounds enhance pigment biosynthesis by facilitating nitrogen assimilation, which is essential for chlorophyll production (Ashraf and Foolad, 2007; du Jardin, 2020; Hare et al., 1998; Van Oosten et al., 2017). As we pointed out, the differential metabolite profiles of these tissues may be associated with the varying effects of leaf and stem powder on maize growth and pigment content. Stems contain a higher concentration of amino acids and development-related metabolites, likely accounting for their significant influence on early development metrics. In contrast, leaves possess greater amounts of flavonoids and pigments, which affect chlorophyll levels.

The biochemical analysis demonstrated notable increases in protein and sugar contents with the 10 g and 15 g treatments, confirming the role of *R. alba* L. powdered biomass in enhancing metabolic activity. Proteins, crucial for enzymatic reactions and structural integrity, exhibited a distinct dose-dependent increase, indicating enhanced nitrogen metabolism in nutrient-rich environments (Ashraf and Foolad, 2007; Calvo et al., 2014; du Jardin, 2020; Eftekharzadeh et al., 2018; Gopalakrishnan et al., 2018; Hare et al., 1998; Rouphael and Colla, 2020; Van Oosten et al., 2017). The accumulation of sugar under these treatments suggests improved photosynthetic efficiency and carbohydrate synthesis, which contribute to energy production and storage for plant growth (McCormick et al., 2008; Ahmad et al., 2024).

Proline is a recognized a stress marker and proline accumulation is frequently induced in plants experiencing stress conditions, such as drought or nutrient deficiency, where it functions as an osmoprotectant (Ashraf and Foolad, 2007; Hare et al., 1998; Liang et al., 2013; Mattioli et al., 2009). The decrease in proline levels following *R. alba* treatment can be interpreted two-fold. The decrease in proline in maize treated with *R. alba* L. may indicate plants which are with a physiological state ready to encounter stress. The findings are consistent with research indicating that plant biostimulants can mitigate various abiotic stresses in different crops (Calvo et al., 2014; du Jardin, 2020; Du Jardin, 2015; Hare et al., 1998; Kanojia et al., 2024). On the other hand, the low proline could indicate over-optimization of the *R. alba* treatment. Future experiments can be conducted to see if *R. alba*-treated maize is really protected against abiotic stresses such as drought and osmotic stress and what is the exact role of proline in it.

The PCA score graphs distinctly differentiate the treated samples from the control, demonstrating a coordinated biochemical response influenced by tissue type and dosage (**Figures 8**, **9**). Chlorophyll a, b, total chlorophyll, and carotenoids significantly influenced PC1, indicating their interrelated functions in photosynthesis and stress resilience (Gitelson et al., 2003; Munné-Bosch, 2005). The sugar content was identified as a significant factor along PC1, underscoring its importance in energy production and resource allocation under treatment conditions. The predominant loading of proline on PC2 indicates that its variation is largely independent of pigment-related traits, supporting the notion that it represents a metabolic adaptation rather than a mechanism for stress reduction. The PCA score graphs distinctly differentiate the treated samples from the control, demonstrating a coordinated biochemical response influenced by tissue type and dosage. Proline primarily contributed to PC2, highlighting its specific function as a stress marker. The distinct alignment suggests that proline accumulation operates independently of photosynthetic and metabolic traits, serving as a specific marker for plant stress conditions (Couée et al., 2006; Szabados and Savouré, 2010). The results highlight the importance of sugars in energy regulation and proline in osmotic adjustment, particularly under varying treatment conditions. However, the stem and leaf powders of *R. alba* influence metabolic processes, as indicated by the PCA loadings. A significant positive correlation exists between photosynthetic pigments and protein with PC1, indicating enhanced biosynthetic activity. The negative loading of soluble sugar in the stem PCA indicates that sugar is primarily utilized for growth-related processes rather than for storage.

The results suggest that *R. alba* L. powdered biomass could function as effective natural biostimulant, providing advantages for sustainable agricultural practices. However, this study did not assess microbial mitigation or breakdown kinetics. Consequently, instead of being recognized as a validated biostimulant effect, the benefits of *R. alba* L. powder could be regarded as responses to growth to soil enhancement. Plant-based treatments improve root development, photosynthetic efficiency, and metabolic stability, suggesting that *R. alba* L. powdered biomass could serve as environmentally sustainable alternatives to synthetic growth stimulants (**Figure 10**). This aligns with the concept of allelopathic cover cropping, in which plant residues and root exudates release bioactive allelochemicals into the soil, influencing crop growth and resource utilization (Kosová et al., 2011). The effectiveness of allelopathic treatments is influenced by environmental conditions, soil characteristics, and treatment concentrations. Climate, organic matter content, and microbial activity are critical factors influencing phytotoxicity levels and nutrient availability (Scavo and Mauromicale, 2021). Therefore, optimizing treatment doses, as demonstrated in this study, is crucial for achieving a balance between growth stimulation and the prevention of nutrient imbalances or toxicity.

## 5 Conclusion

Currently, *R. alba* is used primarily in food industry, medicine, and cosmetics (Gerasimova et al., 2025; Slavov and Chalova, 2024; Valcheva et al., 2024). This research highlights the potential of *R. alba* as a new biostimulant in agriculture, which can enhance the growth of maize, one of the major crops worldwide. *R. alba* itself is an important crop in a few Asian and European countries, notable for its essential oil, and plant biomass from *R. alba* is generated in abundance, it can be utilized for circular bioeconomy purposes to produce large quantities of efficient biostimulant. In the present research, incorporation of powdered *R. alba* leaves and stems into soil enhances the growth rate and physiological functions of *Z. mays* L. during its early stages. The findings indicate that higher powdered biomass doses of *R. alba* biomass, particularly 10 g and 15 g per kg of soil, significantly increased the content of photosynthetic pigments, as well as protein and sugar levels. They facilitated the growth of roots and shoots. The enhancements indicate improved photosynthetic efficiency, heightened metabolic activity, and enhanced nutritional absorption. The powdered biomass ability to alleviate plant stress and maintain physiological stability is significant, as indicated by the reduction in proline levels with higher treatment dosages. Principal Component Analysis (PCA) indicated that sugar content, carotenoids, and chlorophyll positively influenced growth, whereas proline exhibited a negative effect, suggesting reduced stress levels.

Future follow-up studies could further elaborate on the observed growth promoting effect of R. alba. In particular, the following outstanding questions can be addressed: 1) Which of the bioactive *R. alba* compounds (or their combinations) exert the growth promoting effect? 2) Is the biostimulation limited to maize or can be effective to other major crops, and the observed growth and biochemical effects involve nutrient release or mineralization? 3) Which genes and metabolic pathways, in addition to the ones pointed out in this study, are modulated by *R. alba*?

Another important issue is the scalability and affordability of biostimulant products based on *R. alba*, as well as the financial aspects of utilizing such products. In several countries mentioned above, R. alba is a big industry, however focused mainly on producing essential oil and products used in food industry, perfumery, and cosmetics. Here we propose that the plant biomass of *R. alba*, which is currently unused, can be utilized for production of cheap but efficient growth stimulant. Cost benefit and investment return for farmers can be evaluated by further large-scale field experiments. Addressing these questions can help us to understand the fundamental mechanisms behind molecular mode of action of plant biostimulants and encourage the production of inexpensive yet efficient new plant biostimulant for crop improvement.

## Data Availability

The original contributions presented in the study are included in the article/supplementary material. Further inquiries can be directed to the corresponding authors.
